# Explaining combinatorial effects of mycotoxins Deoxynivalenol and Zearalenone in mice with urinary metabolomic profiling

**DOI:** 10.1038/s41598-018-21555-y

**Published:** 2018-02-28

**Authors:** Jian Ji, Pei Zhu, Ivana Blaženović, Fangchao Cui, Morteza Gholami, Jiadi Sun, Jean Habimana, Yinzhi Zhang, Xiulan Sun

**Affiliations:** 1 School of Food Science, State Key Laboratory of Food Science and Technology, National Engineering Research Center for Functional Foods, School of Food Science Synergetic Innovation Center of Food Safety and Nutrition, Joint International Research Laboratory on Food Safety, Jiangnan University, Wuxi, Jiangsu 214122 China; 2 State Key Laboratory of Dairy Biotechnology, Shanghai Engineering Research Center of Dairy Biotechnology, Dairy Research Institute, Shanghai, 200436 China; 3 West Coast Metabolomics Center, UC Davis, 95616 Davis, CA USA; 4 Department of Chemistry, Faculty of Sciences, Golestan University, Gorgan, Iran

## Abstract

Urine metabolic profiling of mice was conducted utilizing gas chromatography-mass spectrometry (GC-MS) to investigate the combinatory effect of mycotoxins deoxynivalenol (DON) and zearalenone (ZEN) on the metabolism of the mice. Experiments were conducted by means of five-week-old mice which were individually exposed to 2 mg/kg DON, 20 mg/kg ZEN and the mixture of DON and ZEN (2 mg/kg and 20 mg/kg, respectively). The intragastric administration was applied for three weeks and urine samples were collected for metabolic analysis. Univariate and multivariate analysis were applied to data matrix processing along with respective pathway analysis by MetaMapp and CytoScape. The results showed that the combined DON and ZEN administration resulted in lower significant changes, compared to the individual mycotoxin treated groups verified by heatmap. Metabolic pathways network mapping indicated that the combined mycotoxins treated groups showed a little effect on the metabolites in most pathways, especially in glucose metabolism and its downstream amino acid metabolism. In glucose metabolism, the content of galactose, mannitol, galactonic acid, myo-inositol, tagatose was drastically down-regulated. Furthermore, the organic acids, pyruvate, and amino acids metabolism displayed the same phenomenon. In conclusion, the combined DON/ZEN administration might lead to an “antagonistic effect” in mice metabolism.

## Introduction

Mycotoxins are secondary metabolites of fungi which are toxic to both human and animals^1^. Yearly, approximately 25% of the world’s agricultural commodities is contaminated by these toxins, especially crops e.g. maize, wheat, barley, millet, peanuts, peas and oily feedstuffs^2^. Fungal infection is a major source of mycotoxins and more than 400 mycotoxins have been identified in 100 fungi strains^3^. Deoxynivalenol (DON) and Zearalenone (ZEN) are recognized as the first two mycotoxins observed in agricultural products, usually co-occurring in food and animal feed^4^.

DON, a trichothecene, is prevalent worldwide in crops used for food and feed production and it is one of the least acutely toxic trichothecenes. DON requires to be considered food safety issues as it is a very common contaminant of grain^5^. Ingestion of DON-contaminated feed can induce anorexia, vomiting, and impaired immune function in various livestock species^6,7^. Previous studies showed that DON could inhibit the protein, DNA, and RNA synthesis leading to cell apoptosis^8^. Animal studies also demonstrated that DON could cause disease or metabolic disorders in kidney, heart, plasma, liver, thymus, spleen and brain^9,10^.

ZEN, mainly produced by fungi, belongs to genus Fusarium genus^11^. It is frequently found in reproductive disorders of farm animals and occasionally in hyperoestrogenic syndromes in humans^12^. There is ab evidence that ZEN and its metabolites incorporate in oestrogenic activity in pigs, cattle, and sheep^13^. Previous reports showed that two estrogen receptor stress-related marker genes, GRP78 and CCAAT/enhancer binding protein homologous protein (CHOP), increased, when treated with ZEN^14^. It might also lead to cell death through p53 or MAPK signaling pathway^15^.

Furthermore, due to the complex process and simultaneous contamination of several mycotoxins in various crops, extremely toxic interactions can potentially occur as a result^16^. However, the toxic effects of the combination of different mycotoxins on animal health and productivity remained uncovered/unrevealed. Unfortunately, the toxicity of mycotoxins in combination cannot always be predicted based upon their individual toxicities^17,18^. The current study increasingly addresses the combined effects of mycotoxins on the metabolism and metabolic pathways changes.

Metabolomics represents the global assessment of metabolites in a biological sample and reports the closest information to the phenotype of the biological system under study^19^. Urine metabolites are the end products of cellular regulatory processes, with the strongest correlation to phenotype, and whose levels can be regarded as the ultimate response of biological systems to genetic and/or environmental changes^20–23^. The present study aimed to utilize a comprehensive GC-MS-based method for urine to evaluate metabolic responses induced by DON and ZEN, in mice. The obtained results will help in understanding the relationship between toxic effects, antagonistic or synergistic toxicity regarding the metabolic profiling assessed.

## Results

### Multivariate analysis of the metabolic profiles

**Figure 1:**
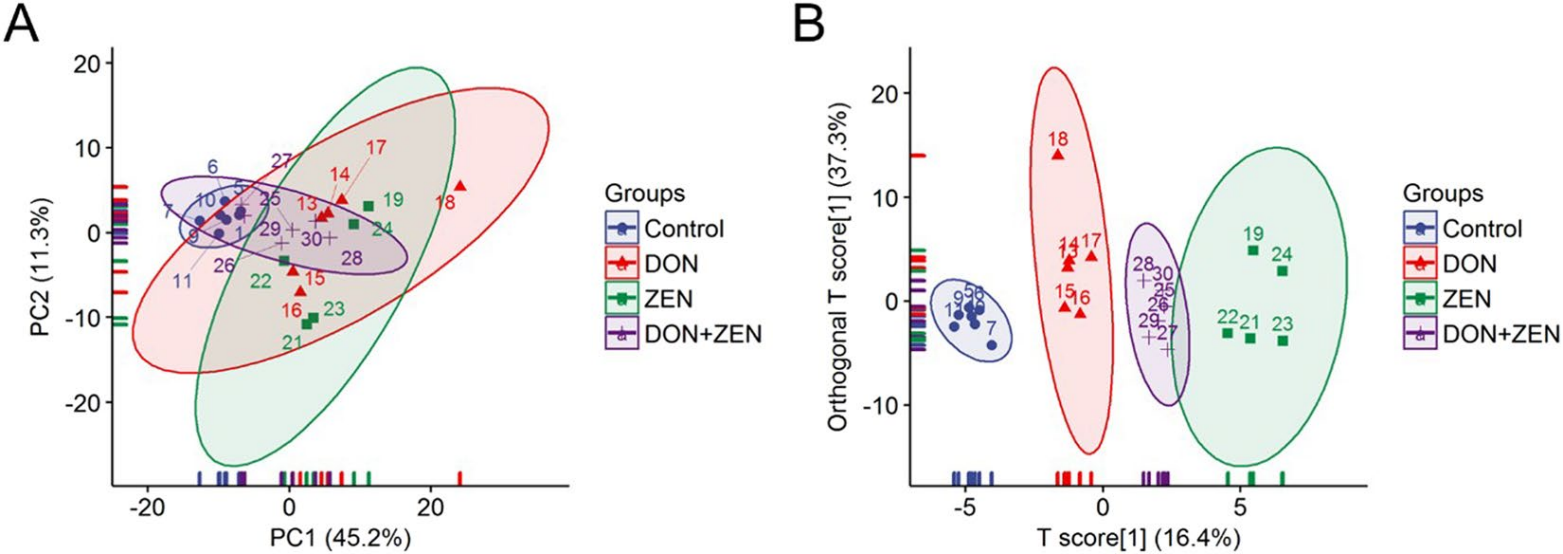
(**A**) PCA and (**B**) OPLS-DA analysis representation of major sources of metabolites variability to monitor metabolites changes in the urine metabolites. Individual DON, (**B**) individual ZEN, and (**C**) combined DON and ZEN. Data points represent urine samples of the three independent experiments (biological replicates; n = 5 ~ 6) injected randomly into the GC-MS. The signals corresponding to different treatments were compared after treatment of log transformation and Pareto scaling.

Metabolite identification process was conducted by comparing the measured mass spectra with standard mass spectra and the retention index in FiehnLib^24^ with MS DIAL software^25^. The raw data (Table S1) were treated with series steps of treatment, including: (1) Removing the unknowns. (2) Dealing with duplicates. The duplicates linear relationship test was applied for validation of one metabolite with multi TMSs after derivatization. If the duplicates have a linear relationship, they will be combined. If there is no linear relationship between the duplicates, the metabolite with lower similarity will be deleted. (3) Removing false positives. The linear relationship between experimental retention time and theoretical retention index (the retention index of metabolites in FiehnLib) was evaluated in order to remove the false positive metabolites. The final data metrics are shown in Table S2. The multivariant statistical analysis was performed by creatinine and mTIC (sum normalization)^26^, data were log-transformed, Pareto scaled; thus, the normal distribution of data (Table S3) was obtained^27^. The PCA score plots (R^2^ = 56.5%) for individual mycotoxins DON and ZEN treated group is represented as a cloud of points in a multidimensional space with an axis for each of the components, which was an unsupervised classification for the displaying the matrix of urine samples. PCA was performed and the score plots of the control and treatment groups are shown in Fig. 1A. Samples in the score plots of identified urine metabolites were within the 95% Hotelling T2 ellipse. As well as the OPLS-DA analysis of identified urine metabolites (R^2^X = 37.3%, R^2^Y = 38.5%, Q^2^ = 30.5%) (Fig. 1B). Outlier detection was done by MetaboAnalyst^28^. Briefly, the function potential outliers under RandomForest analysis was used to recognize and remove the potential outliers, to help improve the quality of data for better separation, prediction or interpretation.

### Heatmap visualization

**Figure 2:**
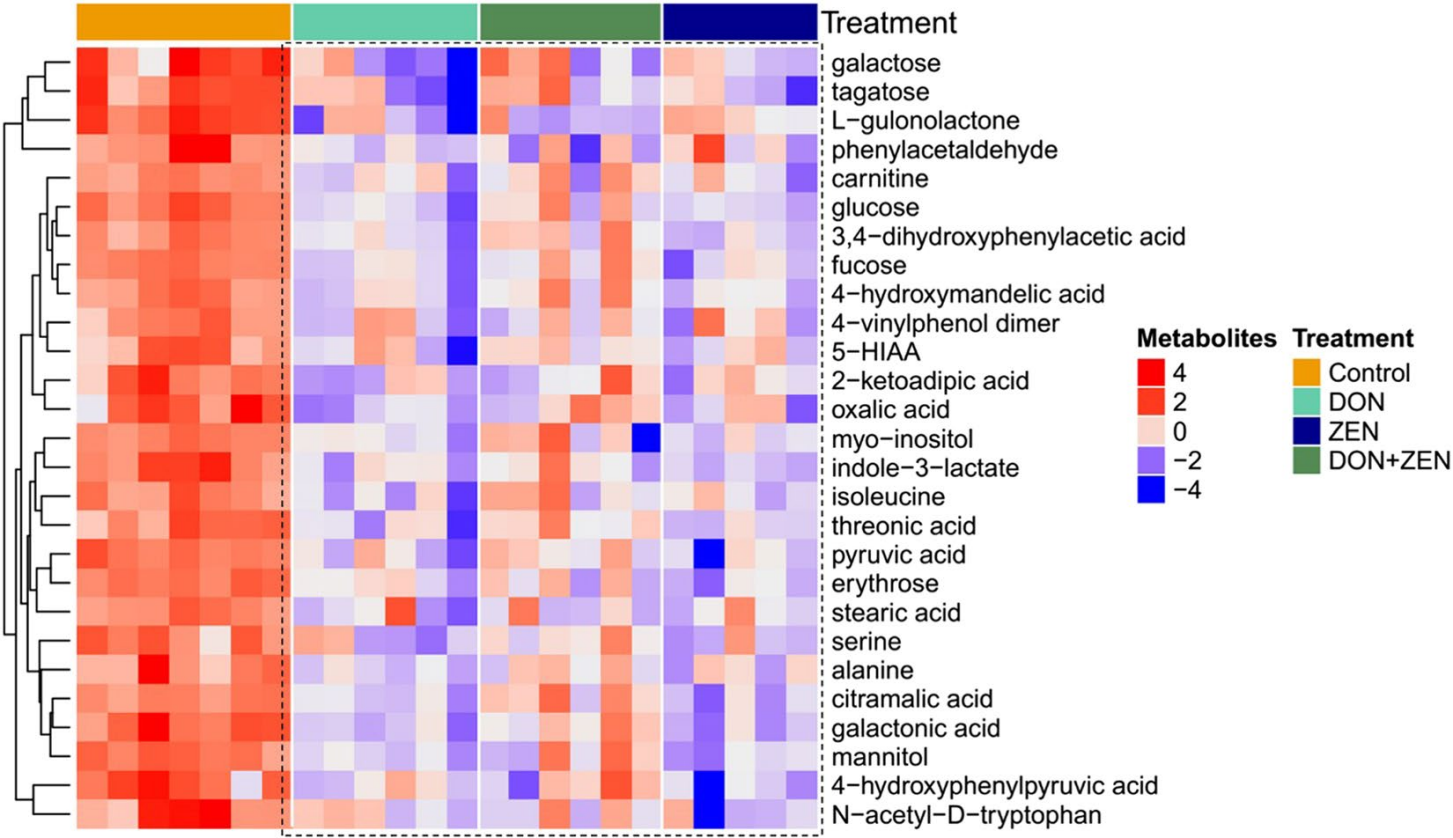
Heat maps, generated by hierarchical Pearson clustering, of selected urine metabolites, significant between the control and three mycotoxins treated groups by ANOVA, FDR p-value < 0.05.

**Figure 3:**
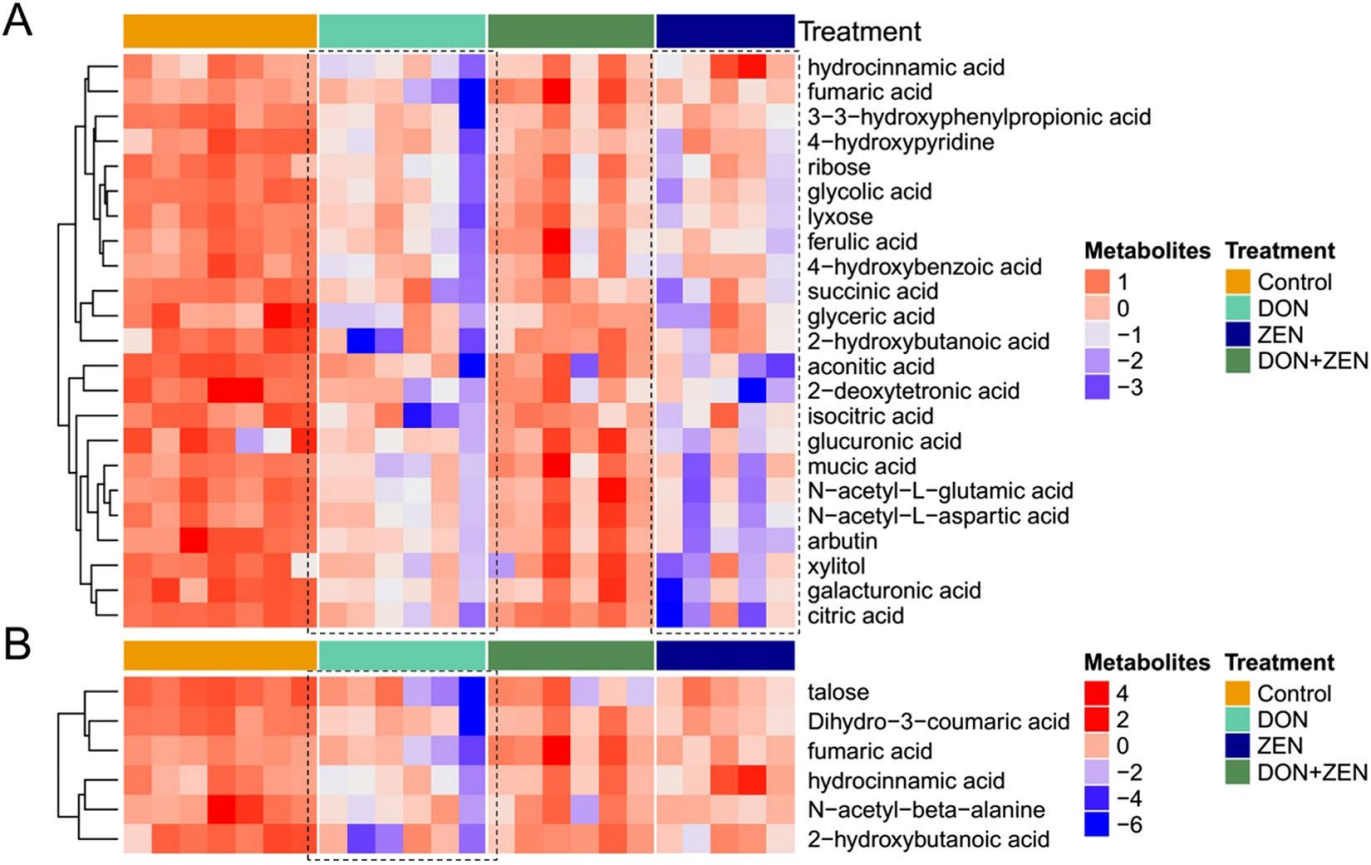
Heat maps, generated by hierarchical Pearson clustering, of selected by ANOVA. (**A**) Urine metabolites, significant between the control and individual DON and individual ZEN groups, non-significant between the control and combined DON and ZEN; (**B**) urine metabolites, only significant between the control and individual DON, FDR p-value < 0.05.

The metabolites selected for heatmap based on the principles of FDR corrected p value (p-value < 0.05) were analyzed by ANOVA test (Table S4). They were split into three groups: the first metabolites group was significant (FDR p-value < 0.05) compared to all mycotoxins treated groups; the second metabolites group was significant (FDR p-value < 0.05) only in individual DON and ZEN groups; the third group of metabolites was significant (FDR p-value < 0.05) only in individual DON group. As shown in Fig. 2, the heatmap was generated by hierarchical Pearson clustering of selected urine metabolites. The results showed a significant difference between the control and three mycotoxins treated groups (FDR p-value < 0.05). Additionally, some metabolites changes were only significant in the individual mycotoxin groups, compared to control group. Twenty-three significant metabolites from both individual DON and ZEN groups demonstrated a decreasing trend (Fig. 3A) and six significant metabolites of individual DON group also revealed a decreasing trend (Fig. 3B).

### Metabolite profiling

**Figure 4:**
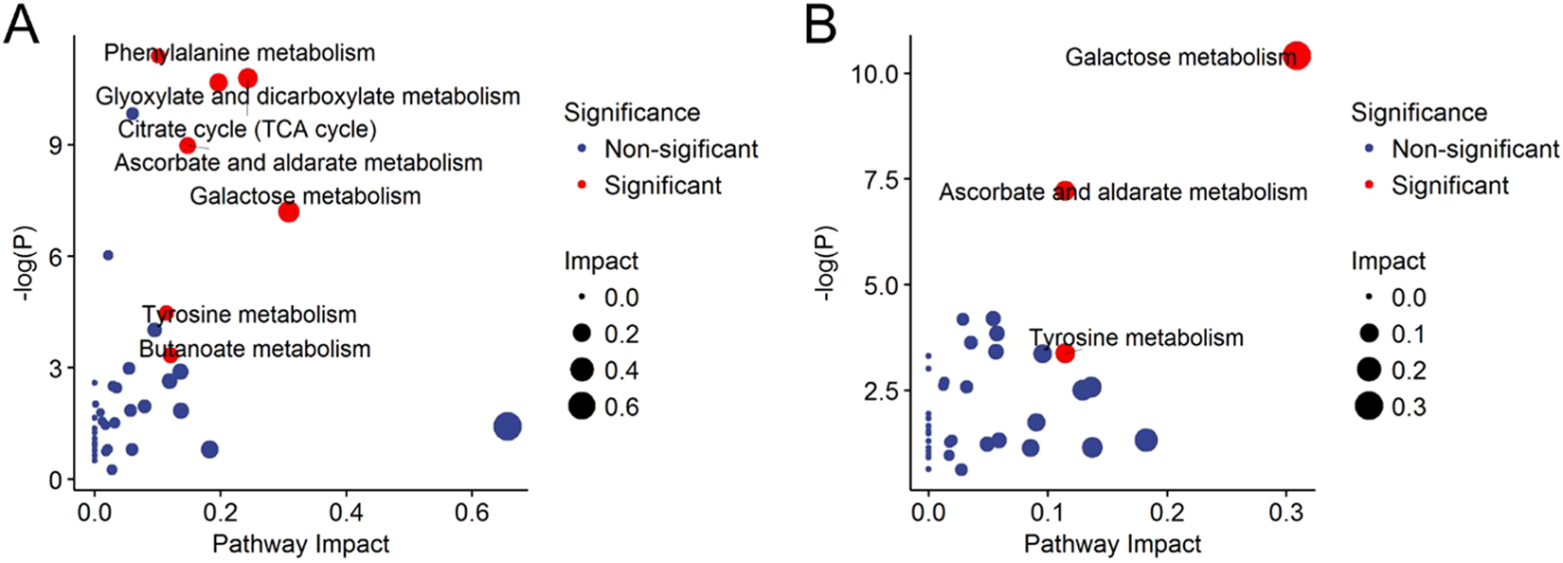
The pathway analysis of the identified metabolites. Based on the selected metabolites, whose FDR p-value < 0.05 between control group with the three mycotoxins dosed groups (**A**) and whose FDR p-value < 0.05 only between control group with individual DON and individual ZEN groups, the global metabolic disorders of the most relevant pathways induced by the MetaboAnalyst 3.0. Here, the x-axis represents the pathway impact and the y-axis represents the pathway enrichment. Larger sizes and darker colors represent higher pathway enrichment and higher pathway impact values.

To better understand the effect of differential metabolism on the individual mycotoxin treated group and combined mycotoxins treated group, the metabolic pathway enrichment were separately analyzed. Firstly, all the significant metabolites of the individual mycotoxin-treated were put into pathway analysis on a web-based tool, MetaboAnalyst 3.0. The pathway analysis algorithms set was a hypergeometric test for over-representation and relative-betweenness centrality for pathway topology analysis, and the final visualized results by R were shown in Fig. 4A, and the entire pathway information is shown in Table S5. Secondly, the significant metabolites in the combined mycotoxin-treated were analyzed, as shown in Fig. 4B, and the entire pathway data is shown in Table S6.

### Integrated pathway analysis

**Figure 5:**
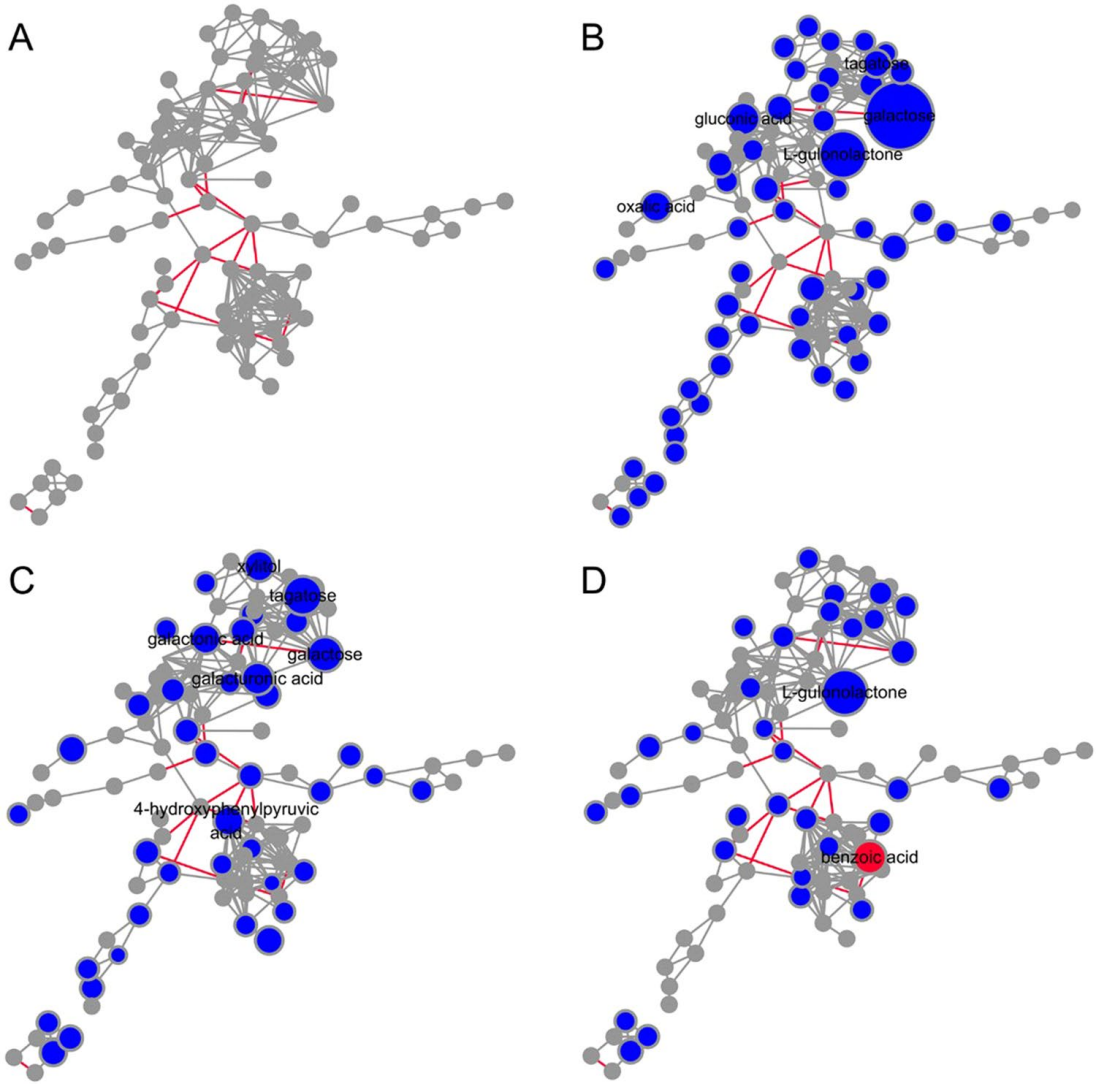
MetaMapp visualization of metabolomic data highlighting the differential metabolic regulation of rats exposed to (**B**) individual DON, (**C**) individual ZEN and (**D**) combined DON and ZEN compared to (**A**) control groups. Red edges denote KEGG reactant pair links; grey edges symbolize Tanimoto chemical similarity at T > 700; unknowns are left out of these graphs for visual clarity. Metabolites found significantly up-regulated under exposure to toxins (p < 0.05) are given as red ball nodes; blue ball nodes give down-regulated metabolites. Grey ball nodes reflect no significance. Only the metabolites, whose fold change value > 10, were labeled for visual clarity.

All the annotated metabolites were included in the integrated pathway analysis, which combined KEGG reactant pairs and Tanimoto chemical similarity tools into a novel method, MetaMapp^29^. In addition, for clarity, the metabolites of the unknown structure have been excluded. Clusters of chemically similar compounds, based on substructure fingerprints within the PubChem database, were used to facilitate biological interpretations. In Fig. 5, 35 KEGG reactant pair links were mapped and 291 PubChem compound pair links were generated based on Tanimoto chemical similarity at T > 700 in MetaMapp. The metabolites relation network was visualized by CytoScape, and the control pathway network was shown in Fig. 5A. In individual DON and ZEN treated mice urine samples, 57% (55/95) of the annotated metabolites were down-regulated fold change direction in individual DON-treated groups and 51% (44/95) of that in individual ZEN treated groups, however, only 34 annotated metabolites were dysregulated in combined DON and ZEN group. As shown in Fig. 5B, all the significantly changed metabolites showed a decreasing trend in the individual DON-treated group. The metabolites, with fold change values > 10, were galactose, tagatose, gluconic acid, L-gulonolactone, and oxalic acid. These are mainly in the galactose metabolism pathway, and are consistent with the pathway enrichment result. As shown in Fig. 5C, in the individual ZEN-treated group, the metabolites with fold change values > 10, including gluconic acid, citric acid, tagatose, galactose, galacturonic acid, xylitol, galactonic acid, 4-hydroxyphenylpyruvic acid, were mainly incorporated in the galactose metabolism and TCA cycle. It could be observed that a small number of metabolites decreased obviously and metabolite benzoic acid significantly increased (Fig. 5D). It was also obvious that the red-colored nodes in the networks of combined DON and ZEN group were less than that in individual DON or ZEN group. The metabolites with fold change values > 10 including L-gulonolactone, benzoic acid, mainly of the phenylalanine metabolism pathway. MetaMapp was applied for the integrated metabolic pathways analysis, and the output is shown in Table S7 and Table S8. Fold change direction was calculated in MetaMapp based on two factors, the *t-test* p-value and fold change value.

## Discussion

The mycotoxins doses were selected based on previously published research^30,31^. Briefly, 2 mg/kg DON, 20 mg/kg ZEN and the mixture of DON and ZEN (2 mg/kg DON, 20 mg/kg ZEN), were implemented to mice through intragastrical administration for three weeks, referring to the duration in sub-chronic toxicity test^32^, as well as the change of physical and chemical indicators in mice.

Distinct spectral phenotypes were readily observed in the identified metabolites of the urine samples collected from control and mycotoxins treated mice. The observed difference between the mycotoxins treated and control groups, either individual or combined mycotoxins, indicates the inconsistency of metabolic profiles and implies that the individual mycotoxin might lead to more severe damage on the mice bio-system.

The heatmap visualization of those metabolites, showing significant difference in the all mycotoxins treated groups, revealed that all the selected metabolites decreased in the three treated groups, indicating that the metabolism pattern was different between individual mycotoxin groups and the combined mycotoxins group, consistent with OPLS-DA results. The hierarchical Pearson clustering analysis for individual DON and ZEN treated groups revealed a significant difference between the dose and control groups, in consistent with PCA result.

Metabolic pathway analysis was investigated for further insight into urine disorder metabolic function in urine. A number of metabolism pathways including galactose metabolism, citrate cycle (TCA cycle), glyoxylate and dicarboxylate metabolism, ascorbate and aldarate metabolism, butanoate metabolism, tyrosine metabolism, phenylalanine metabolism, were affected by individual mycotoxins. Besides, in the combined mycotoxins treatments, ascorbate and aldarate metabolism, galactose metabolism, and tyrosine metabolism were of the three metabolic pathways ranked as significant (pathway effect value > 0.1).

As metabolites p and fold change values are not taken into consideration by MetaboAnalyst, the pathway results could not comprehensively represent the metabolism changes. An integrated pathway analysis was required to descript the global metabolic disorder. To better understand metabolic disorders discrepancy between the individual mycotoxin-treated groups and combined mycotoxin-treated group, an integrated pathway analysis was implemented. MetaMapp is an R based script whose outputs are seamlessly compatible with the open-source platform CytoScape, visualization of next-generation metabolomics datasets with an increased number of identified metabolites^29^. It can be used to visualize all detected metabolites in metabolomics studies that can comprise both identified and unknown compounds while maintaining the modular organization of metabolites in biochemical pathways^33^.

**Figure 6:**
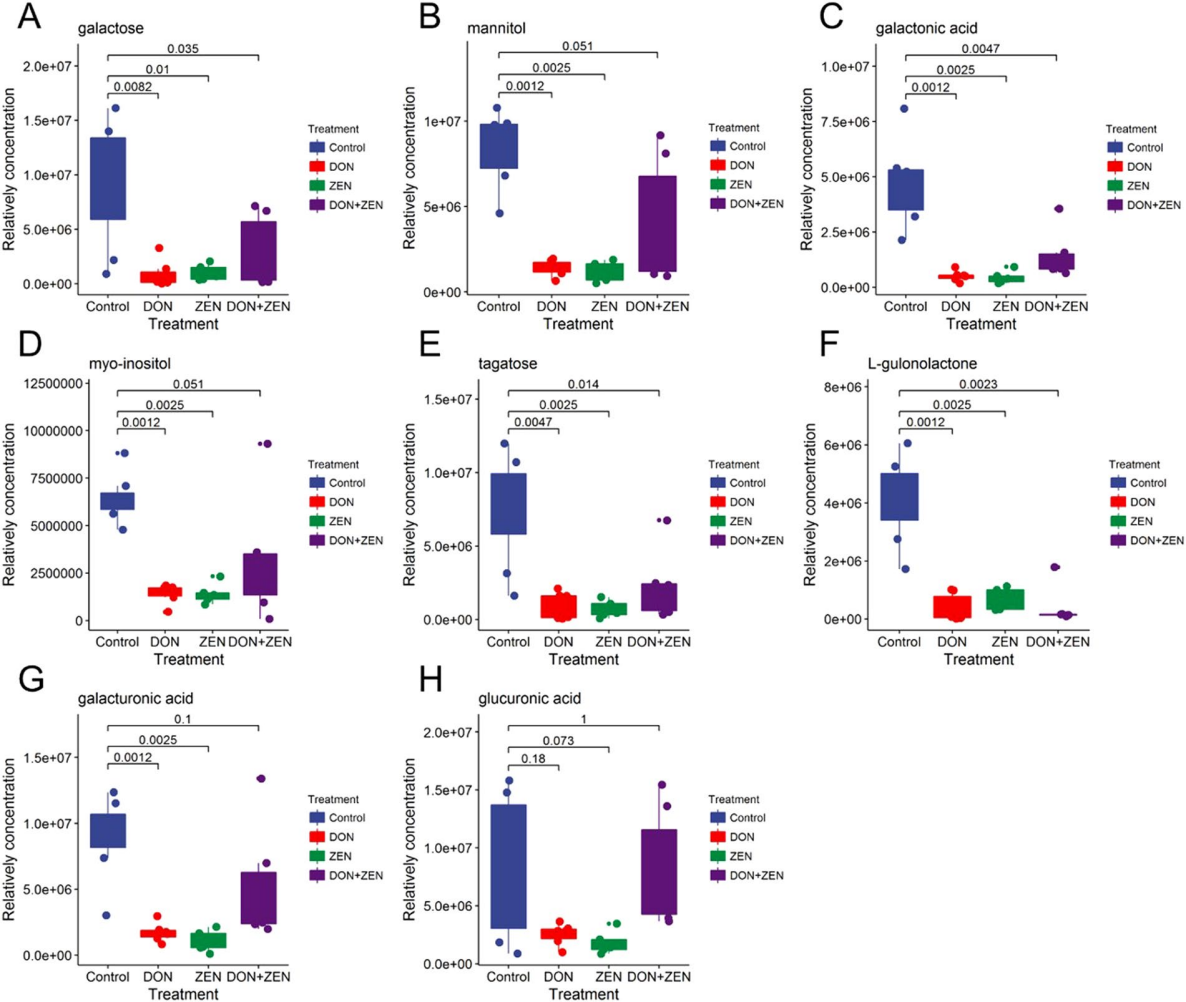
The peak height comparison of glucose derivative metabolites in individual DON group, individual ZEN group, combined DON and ZEN group, and control group. The Dunnett’s multiple comparison tests were implemented for statistical analysis of the significance between the treatment group and the control group. Raw p-value was labeled.

Glucose is used as main energy source in cells by either aerobic respiration, anaerobic respiration, or fermentation^34^. These processes follow from an earlier metabolic pathway known as glycolysis. However, the glucose derivative or glucose isomers also play a decisive role in glucose metabolism, refining to galactose metabolism^35^, glyoxylate and dicarboxylate metabolism^36^, glycerolipid metabolism^37^. The glucose derivative or glucose isomers, which had meaningful changes in the mycotoxins dosed groups were integrated into one metabolic pathway. To investigate the combinatory effect of mycotoxins, DON and ZEN, on glucose metabolism in mice, the peak intensity of these carbohydrate compounds was displayed in boxplot of the control group and three mycotoxins dosed groups, to investigate the combination effect of mycotoxins, DON and ZEN, on glucose metabolism in mice. In individual DON or ZEN group, galactose and its downstream metabolites, including mannitol, galactonic acid, myo-inositol and tagatose displayed more significant down-regulation, compared to the combined DON and ZEN group. Both of individual mycotoxins and combined mycotoxins groups influenced the glucose metabolism, however, individually dosed manner displayed a more serious effect, as shown in Fig. 6, L-gulonolactone, or it might be called an “antagonistic effect” in the “biological metabolism” of mice. Galacturonic acid and its downstream metabolites such as glucuronic acid are both significantly decreased in the individual mycotoxins treated groups but only slightly decreased in the combined group. In our previous study, some specific canonical markers were detected, like blood urea nitrogen, creatinine, alkaline phosphatase (ALP), alanine transaminase (ALT), total protein (TP), albumin (ALB), globulin (GLO), ALB/GLO in serum, which indicated the antagonistic effects between DON and ZEN^38^. The antagonistic effects also existed in serum metabolism and some tissue metabolism, including liver, kidney, spleen^18^.

**Figure 7:**
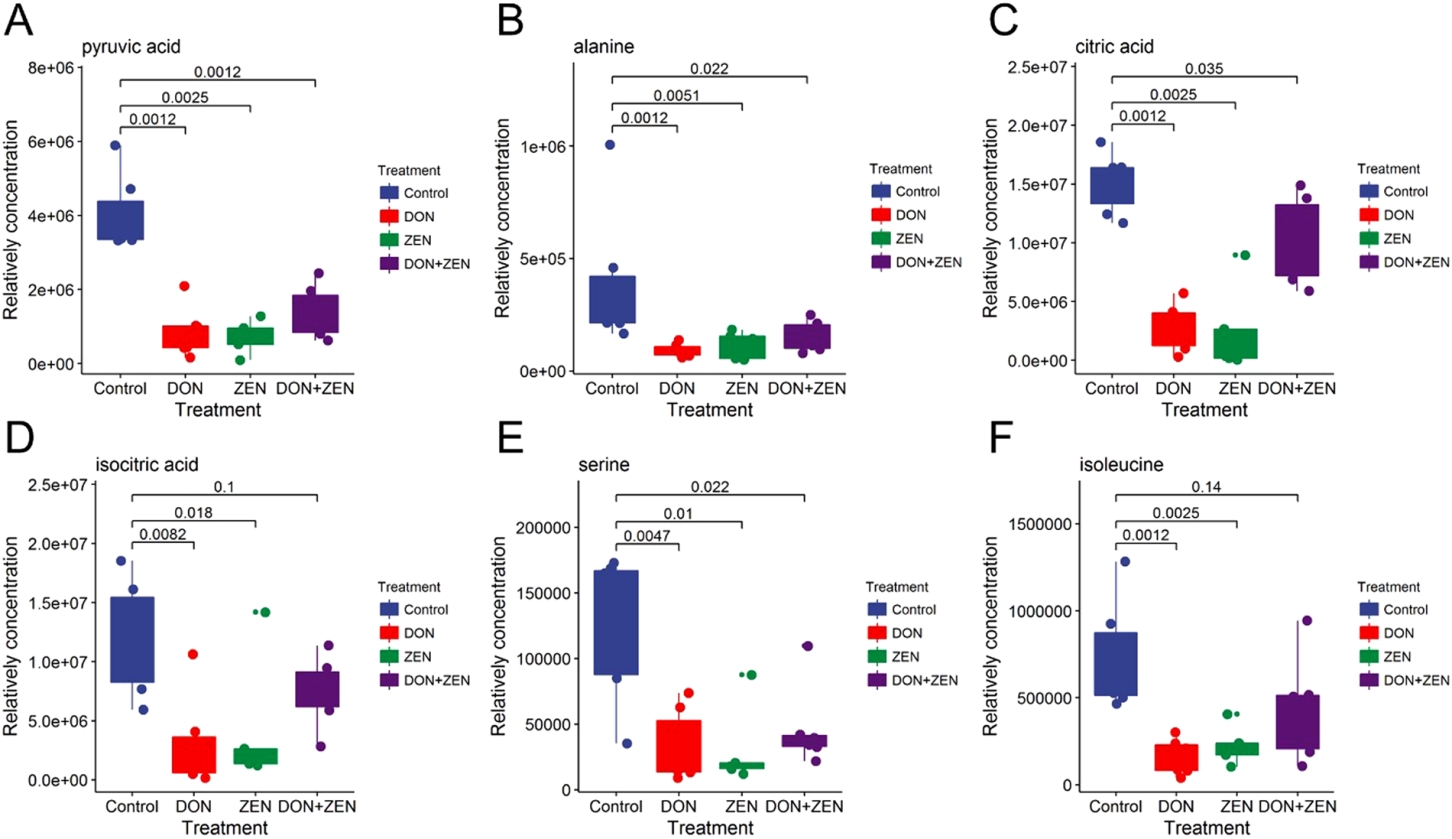
The peak height comparison of amino acids in individual DON group, individual ZEN group, combined DON and ZEN group, and control group. The Dunnett’s multiple comparison tests were implemented for statistical analysis of the significance between the treatment group and the control group. Raw p-value was labeled.

Glycolysis is the metabolic pathway through which glucose converts to pyruvate which is further metabolized to amino acid alanine via transaminases. Furthermore, pyruvate can be converted to ethanol or lactic acid during fermentation^39^. As shown in Fig. 7, alanine content undergoes a significant decrease in individual DON and ZEN treated groups. In combined mycotoxins treated group, pyruvate displayed a serious down-regulated trend, however, the other three amino acid performed slightly decreasing tendency. These compounds including amino acids (serine, isoleucine, and alanine) and organic acids (isocitric acid and citric acid), are involved in the integrated amino acid pathways, mainly in TCA cycle.

**Figure 8:**
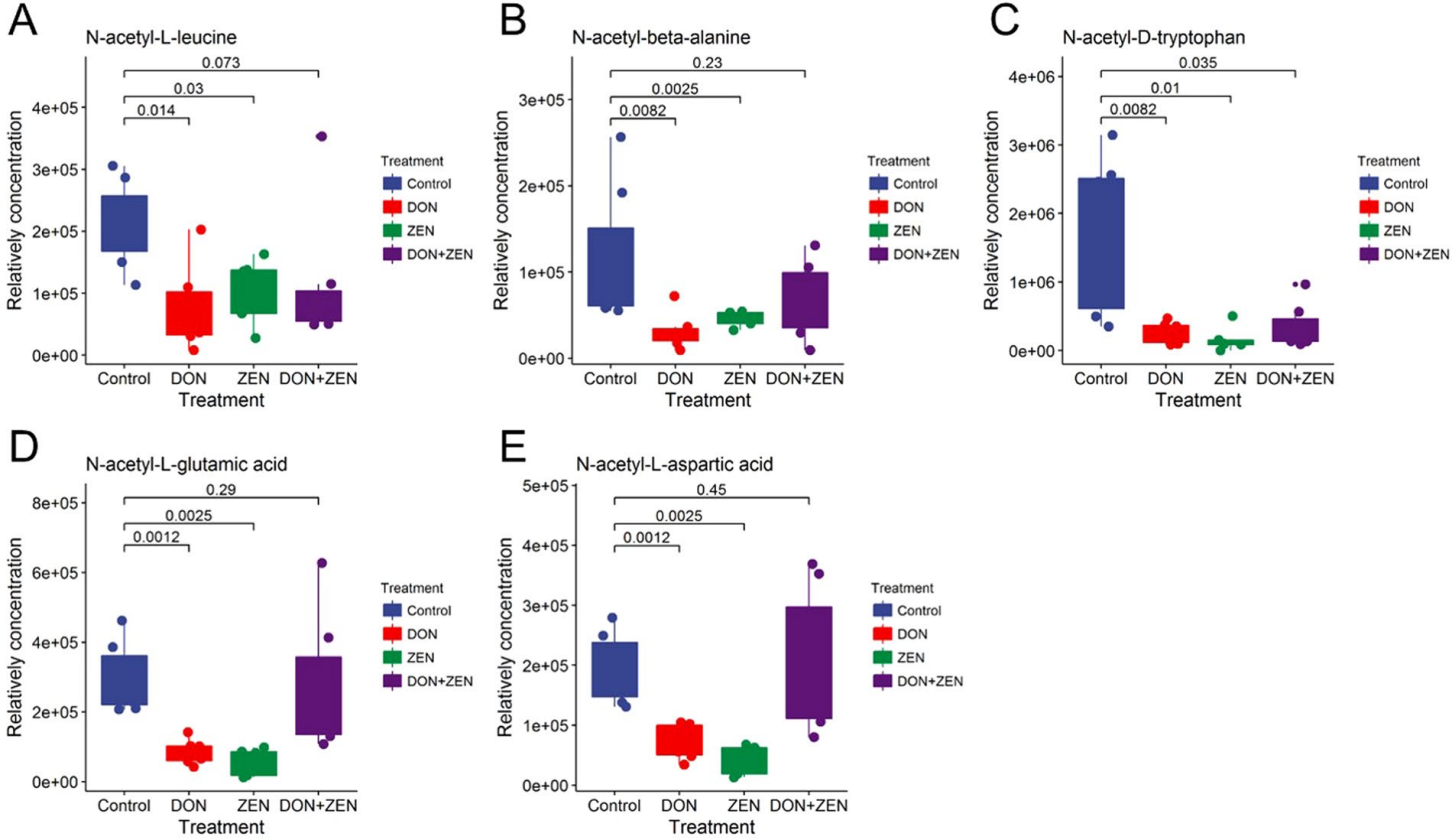
The peak height comparison of acetylated amino acids in individual DON group, individual ZEN group, combined DON and ZEN group, and control group. The Dunnett’s multiple comparison tests were implemented for statistical analysis of the significance between the treatment group and the control group. Raw p-value was labeled.

As shown in Fig. 8, the acetylated amino acids, including N-acetyl-L-leucine, N-acetyl-glutamic acid (NAcGlu), N-acetyl-L-aspartic acid, N-acetyl-D-tryptophan, and N-acetyl-beta-alanine, were usually biosynthesized from specific amino acid and acetyl-CoA by the enzyme N-acetyl-(amino acid) synthase^40,41^. NAcGlu activates the carbamoyl phosphate synthetase in the urea cycle^42^. The decreasing trend of N-acetyl amino acids may be the result of a deficiency in N-acetyl amino acids synthase or a genetic mutation in the gene coding for the enzyme, which could lead to metabolic disorders or disease^43,44^. For instance, the lack of NAcGlu will lead to urea cycle failure in which ammonia is not converted to urea and accumulates in the blood causing Type I Hyperammonemia^45^. Furthermore, N-acetyl-L-aspartic acid, whose various functions are still under investigation, has the primary proposed functions to certain lipids, myelin, and neuronal dipeptide synthesis^46^. The decreased trend of acetylated amino acids in individual mycotoxins DON and ZEN group concludes the “antagonistic effect”.

## Conclusion

Urine metabolomics was conducted to investigate the combined effects of DON and ZEN in mice bio-system. The combined DON and ZEN could lead to an “antagonistic effect” mainly in galactose metabolism and TCA cycle. Glucose metabolism showed a decreasing trend of galactose and its downstream metabolites, including mannitol, galactonic acid, myo-inositol, and tagatose along with decreasing trend of pyruvate, isocitric acid, serine, isoleucine, citric acid and alanine, as well as their acetylated amino acids.

## Methods

### Chemicals and reagents

N-Methyl-N-(trimethylsilyl)trifluoroacetamide (MSTFA), deoxynivalenol (DON), zearalenone (ZEN) and methoxyamine hydrochloride, were purchased from Sigma-Aldrich Co. LLC., USA. The phosphate buffer solution (PBS, 0.1 M K_2_HPO_4_/NaH_2_PO_4_, pH 7.29) used was purchased from Beyotime biotechnology, China. All other chemicals used were of HPLC grade. Deionized water used for all experiments was purified with a Milli-Q system (Millipore, USA).

### Animal handling and treatment

The ethical guidelines of the European Community guidelines were applied to ensure the experimental animals received humane care (Directive 2010/63/EU), and all the experimental procedures were approved by the Jiangsu Science and Technology Department (SYXK[Su]2012–0002) with the document signed on 7 April 2016, project ID: JN.NO.20160322–20160701[25]. 50 Kunming (KM) mice (25male and 25 female), five-week age, were purchased from the Shanghai experimental animal facility. All the mice were acclimatized in polypropylene cages at room temperature, relative humidity of 50 ± 10%, light cycle maintained at 12 h of light and 12 h of darkness for 7 days. Food and water were provided *ad libitum*. 40 mice were randomly separated into 4 groups, 8 cages with an equal number of animals (n = 10 in each group, 5 male mice and 5 female mice in 2 cages, feeding separately) treated with 2 mg/kg DON, 20 mg/kg ZEN and the mixture of DON and ZEN (2 mg/kg DON, 20 mg/kg ZEN), through intragastric administration for three weeks. The solvent used for dilution of mycotoxins was 10% ethanol and 90% saline. The mice of the control group were treated with solvent.

### Urine collection and extraction of metabolites

Urine samples were collected in glass cuvettes and centrifuged within 1 h at 3000 rpm for 10 min at room temperature. The supernatant was aliquoted into Eppendorf tubes with 1 mL urine in each and stored at −80 °C until use. Urine samples were thawed at room temperature for about 15 minutes. 50 μL of urine was then aliquoted on ice. 50 μL urease aliquot (40 g/L) was added to each urine sample following the shaking for 1 h at 37 °C to reduce the risk of column overloading, peak distortions or matrix effects, and ion suppression for GC–MS analysis^47^.

Metabolites extraction followed the SOP of Fiehn lab^48^. Briefly, urine samples were centrifuged under 13,000 × g at 4 °C for 15 min, 50 μL supernatant urine was mixed with 1 mL extraction solution (acetonitrile/isopropanol/H_2_O (3:3:2, v/v)). After vortexed for 10 s, the mixture was centrifuged at 13,000 × g at 4 °C for 15 min. 300 μL supernatant was collected in EP tube. Quality control: An equal volume of 10 μL was collected from each sample into the 2 mL GC-MS glass vial as a sample pool^49^. Samples and QCs were dried for six hours in the Savant High Capacity Speedvac Plus Concentrator (Thermo Fisher, USA). 80 μL of 20 mg/mL methoxylamine hydrochloride was added into dried metabolites followed by incubation at 80 °C for 20 min in an oven after vortex. 100 μL MSTFA (containing 1% TCMS, v/v) was added to each sample, vortexed and incubated at 70 °C for 60 min. Centrifugation with the mentioned parameter, the 700 μL supernatant were ready for GC-MS analysis^50^.

### GC-MS analysis

Shimadzu QP2010 Ultra gas chromatograph system coupled with a mass spectrometer was applied for GC-MS data acquisition. The system utilized a Rxi-5Sil MS column (30 m × 250 μm inner diameter, 0.25 μm film thickness; Restek, USA). Injection volume: 1 μL, with splitless injector; carrier gas: Helium; the front inlet purge flow: 20 mL min^−1^; initial temperature: 70 °C for 1 min, then raised to 280 °C at a rate of 6 °C min^−1^, and maintained for 5 min at 280 °C. Injection temperature: 280 °C; transfer line temperature: 280 °C; ion source temperature: 250 °C. Electron impact mode energy: −70 eV; solvent delay: 366 s; full-scan mode range: 50~600 m/z; scan rate: 20 spectra per second.

### Metabolite profiling

Shimadzu GCMS PostRun software was used to convert the raw data to “mzXML” format, then converted to “abf” format with the ABF converter. The MS DIAL equipped with FiehnLib^24^ was used for raw peaks exaction, peak alignment, deconvolution analysis and identification, *et al*.^25^. Parameter settings were as follows: average peak width: 20; scan and minimum peak height: 10000; sigma window value: 0.5, EI spectra cut off: 5000. The retention time tolerance: 0.5 min, the m/z tolerance: 0.5 Da, the EI similarity cut off: 70%, the identification score cut off: 70%. Alignment settings: retention time tolerance: 0.075 min, retention time factor: 0.5. The raw data is accessible in Table S1.

### Multivariate analysis

Principal component analysis (PCA), orthogonal projection to latent structures-discriminant analysis (OPLS-DA) analysis and pathway enrichment were analyzed on Metaboanalyst 3.0, a web-based tool for metabolomics pathway analysis. The visualization of PCA, OPLS-DA, heatmap analysis and pathway enrichment were performed with R i386 3.4.1. The pathway mapping was analyzed with MetaMapp and CytoScape. The statistical analysis, including one-way ANOVA test (Dunnett’s multiple comparison tests) and *t-test*.

### Data availability

Data files are accessible through the MetaboLights, a public metabolomic repository. The study identifier is MTBLS580. https://www.ebi.ac.uk/metabolights/MTBLS580.

## Electronic supplementary material

Dataset 1

Dataset 2

Dataset 3

Dataset 4

Dataset 5

Dataset 6

Dataset 7

Dataset 8
